# Iodide of Potassium in Syphilis

**Published:** 1878-12

**Authors:** J. F. Miner


*ART. II.—Iodide of Potassium in Syphilis.—Clinical Remarks.* By Prof. J. F. MINER. Reported with private Surgical Cases by C. A. WALL, B. S., a member of the Class.

GENTLEMEN: I present before you, first this A. M., a man sent here for the operation for Tracheotomy—Syphilitic Laryngitis—having so completely interrupted respiration that on entering the Hospital three days ago it seemed impossible for him to live until the time of our clinic, unless immediately relieved; but as the dyspnœa had come upon him gradually, and he had become accustomed to it, he himself thought he could wait until this morning. You will observe he has long been a victim of syphilis; the bones of the nose have necrosed and been discharged—allowing of the deformity, you observe so characteristic of syphilitic necrosis of the bones of the nose. The odor of this patient is also pathognomonic of syphilis. He has been under treatment by various physicians three years, with the results you observe. Possibly he has been too poor to purchase proper medicine for his disease if it has really been prescribed for him. His condition is pitiable in the extreme, and his physicians have sent him here as a last resort to get rid of him, of course, and have recommended tracheotomy.

Tracheotomy was first suggested by Prof. Rochester, and I have regarded it as offering the only possible opportunity to fully test the value of proper medication--of testing what medicine can do for syphilis, as without relief he must soon die of suffocation. Upon his entrance I advised 20 grs. iod. potass. three times daily, proposing to increase it until satisfactory results followed; but I did not expect to see so great relief in so short time. He now breathes with comparative comfort, and is willing to try still longer what medicine will do for him; and though I had no doubt of the necessity of tracheotomy three days ago, I this morning think it may be avoided. His voice is now so restored that he articulates quite distinctly. His countenance has changed, so that he looks cheerful and hopeful, instead of the expression of despair, characterizing the feeling of strangulation. You would like to witness the operation of tracheotomy, but you will appreciate how much more valuable the lesson. If I could select from the world an interesting and instructive case in Operative Surgery (?) for your observation, I would select this most signally successful operation of medicine in syphilis. He is pale, thin, weak, dejected—ready for any operation offering even temporary relief. He is deformed by his disease, in constant pain, preventing sleep and rest. He catches at the last straw of tracheotomy, thus hoping to gain time for better effects of medicine. Twenty grains is a small dose compared with what, if necessary, it is proper to prescribe. Mercury is a specific in the secondary stage of syphilis. Iodine—Iodide Potassium—is equally specific in the tertiary form.

I have not yet described to you the stages of syphilis, but most of you understand my meaning. Mercury and Iodine properly prescribed are curative of syphilis. Since our term commenced I have been called to advise in several cases as nearly hopeless as this before you, and in every case the effects of *adequate* doses of Iodide of Potassium was as signally successful. One of our citizens says he would have been willing to have paid $50,000 for the advice if he could thereby have saved his deformity. Caries of the bones of the nose is common in syphilis—is to be watched for and dreaded. And, gentlemen, after adequate experience I can assure you that almost always it can be prevented, and when present *can be cured.*

Ah! That word *cure* is a bad word. If you use it much it will show that you are a quack. Good, honest, intelligent physicians rarely have place for it, but it is more truthfully used in syphilitic disease than anywhere else in medicine, though a distinguished Vienna Professor says to his class: "If a patient contract syphilis, talk of his *cure* as much as you like, he will die syphilitic, and his soul will come up in the judgment with syphilis." Which simply means, it cannot be wholly removed from the general system so as not to be liable to again appear. As to its being extended to the soul, I am not so clear. Many syphilitic patients will appear in the judgment with as pure a soul as will ever go from earth to heaven and I do not think the Vienna Professor designed anything more than that *syphilis is incurable.*

I must not detain you longer than to say that *adequate* doses of Iodide of Potassium will almost invariably arrest and control syphilis in its tertiary form, and by *adequate* I mean from twenty to one hundred grains given three times daily, where smaller quantities do not answer the purpose. This looks heroic, but it is safe and often very desirable; and given when the stomach contains food or fluid it does not cause distress or other inconvenience. It may produce the condition called Iodism, but continue, or, if necessary, *increase* your medicine. Such patients have a thousand times more to fear from syphilis than "iodism," and the iodism will soon pass off itself.

Tell your associate physicians what I say of this, and many of them will think I am insane on this subject, but I know of what I speak. I speak as one having "authority and not as the scribes." Ample experience convinces me I am correct in this conclusion of the proper dose of Iodide of Potassium, and you may safely adopt it until experience teaches you that it is *unnecessary* or *unsafe.* I am sustained in all this by some of the best authors, though differences of opinion are freely expressed by others. And, you need not decide this morning, where "Doctors disagree."

P. S. (One week later.)

Gentlemen.—I am now able to show you the final result in our patient, who was so greatly relieved by the Iodide of Potass, and was before you at our last Clinic. He continued comfortable until yesterday A. M., but was observed to grow rapidly worse through the day. House Physicians observed the rapidly increasing dyspnœa, but until night thought our Clinic would be ample time for interferance should any be advisable. At 12 o'clock, night, they saw he was rapidly failing and sent message for me. In a few minutes more before my arrival he was dead, and we propose to show you the changes in the Larynx, which have resulted in death. (After dissection) you will observe as I pass this specimen, how all the inner surface is thickened and tube closed. All this thickened ulcerated condition we expected to find, but we also observe a necrosed piece of bone, furnished by ossification of the anterior laryngeal cartilage. This dead bone is the effect of Syphilis, as you remember the nasal bones have preceded this one and escaped. Others were following the same course. Tracheotomy as proposed at our last Clinic, would have been proper, might have given time for action of remedies and have prolonged life. The specimen is instructive in the highest degree; it shows you what awful changes syphilis is capable of producing and how it may be the direct cause of death.

Case IX.—Eddie ——, four years of age, from German parentage, is deformed in both hands, but as the left seemed to present the most favorable condition it was operated upon June 24th, 1878. The little finger and thumb were normal, while the middle finger was wanting, the head of the corresponding metacarpal bone articulating with a peculiar compound bone which was joined with the index and ring fingers. The bone was V shaped and formed by three centers of ossification, two for the ring finger portion and one for the index. The former was one and one-half inches in length, the latter one inch. The shape of the bone rendered it impossible to approximate the ring and index finger. The difference between the heads of the first phalanx of each finger was two inches. This peculiar bone was removed, the parts brought together with interrupted sutures and a bandage thrown around the hand. It soon healed and the hand is very useful, perfect in all but the middle finger. The right hand was not operated upon. The thumb presented two sets of phalanges, parallel but frail, and neither set was strong enough to give the necessary strength to the thumb.

CASE X.—*Dislocation of the ankle outwards.* March 26th, 1878, Emma S., aged eight, while playing upon a pile of lumber slipped, carrying a number of pieces with her. The weight of these fell upon her ankle, and as both the foot and leg were supported the force was sufficient to dislocate the ankle outwardly. She was immediately released, and in less than twenty minutes after the accident the ankle was returned to its place. There was little or no exudation, and no signs of serious injury to ligamental structures. The next day she was hopping around without pain, and in two weeks had forgotten which foot had been hurt. There was no doubt as to the dislocation as the foot lay at right angles to the leg, and would not yield until after considerable force had been applied. This is a rare case of dislocation, ranking with those of the wrists. Dr. Miner presented to the Buffalo Medical Association a case of the latter, (see Buffalo Medical Journal, Vol. XVIII, No. 3, p. 95), in which it was mistaken for a Colles fracture.

